# Three-dimensional, symmetrically assembled microfluidic device for lipid nanoparticle production†

**DOI:** 10.1039/d0ra08826a

**Published:** 2021-01-05

**Authors:** Niko Kimura, Masatoshi Maeki, Kosuke Sasaki, Yusuke Sato, Akihiko Ishida, Hirofumi Tani, Hideyoshi Harashima, Manabu Tokeshi

**Affiliations:** Graduate School of Chemical Sciences and Engineering, Hokkaido University Kita 13 Nishi 8, Kita-ku Sapporo 060-8628 Japan +81-11-706-6745 +81-11-706-6744; Division of Applied Chemistry, Faculty of Engineering, Hokkaido University Kita 13 Nishi 8, Kita-ku Sapporo 060-8628 Japan tokeshi@eng.hokudai.ac.jp m.maeki@eng.hokudai.ac.jp +81-11-706-6745 +81-11-706-6745; JST PRESTO 4-1-8 Honcho, Kawaguchi Saitama 332-0012 Japan; Faculty of Pharmaceutical Sciences, Hokkaido University Kita 12 Nishi 8, Kita-ku Sapporo 060-0812 Japan; Innovative Research Center for Preventive Medical Engineering, Nagoya University Furo-cho, Chikusa-ku Nagoya 464-8601 Japan; Institute of Nano-Life Systems, Institutes of Innovation for Future Society, Nagoya University Furo-cho, Chikusa-ku Nagoya 464-8601 Japan

## Abstract

Sub 100 nm-sized lipid nanoparticles (LNPs) have been widely used in drug delivery systems (DDSs). The size of the LNPs is an important parameter for the DDS performance, such as biodistribution and gene silencing using siRNAs. However, the LNPs prepared by the conventional preparation method show a wide size distribution. To improve the LNP size distribution, we developed a microfluidic device, named the iLiNP™ device, in a previous study. This device could produce LNPs in the size range of 20 to 150 nm, but the size distribution of the large-sized LNPs needs to be further improved. From the viewpoint of the LNP formation process, a homogeneous and slow rate dilution of ethanol plays an important role in improving the large-size LNP size distribution. In this study, we developed a three-dimensional, symmetrically assembled microfluidic device named the 3D-iLiNP device with the aim of precise size control of large-sized LNPs. We designed the 3D-iLiNP device using a computational fluid dynamics simulation and demonstrated that the 3D-iLiNP device can improve the LNP size distribution. The gene silencing activity of four kinds of siRNA-loaded LNPs was investigated *via in vitro* and *in vivo* experiments to elucidate the effect of the LNP size distribution. The results revealed that the LNPs with a size between 90 and 120 nm showed higher gene silencing activity than those with other sizes. The 3D-iLiNP device is expected to improve DDS performance by precisely controlling the size of LNPs.

## Abstract 2

We developed a three-dimensional and symmetrically assembled microfluidic device named the 3D-iLiNP device. The 3D-iLiNP device allowed the precise size control of sub-100 nm sized lipid nanoparticles by the homogeneous and slow ethanol dilution.

## Graphical abstract

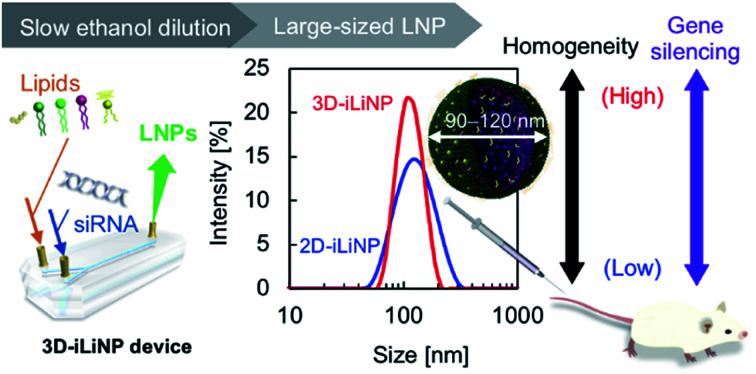

## Introduction

Lipid nanoparticle (LNP)-based drug delivery systems (DDSs) are among the most advanced nanomedicine systems. The size of LNPs plays an important role in both the LNP biodistribution and LNP performance, such as antitumor effect and gene silencing activity.^1–7^ Generally, small-sized LNPs are desirable for stromal rich tumor tissues, such as pancreatic cancer. On the other hand, 100 nm-sized LNPs can deliver drugs or RNA into the hepatocytes of liver tissues passing through the fenestrae (with an approximate diameter of 100–150 nm).^8^ Recently, the relationship between the LNP size and DDS performance has been investigated widely (Table S1†). In previous studies, this relationship was investigated with LNPs with a size of 10–50 nm. However, LNPs are produced with a size distribution in spite of size tuning such as extrusion using a polycarbonate filter and ultra-sonication.^1,9^ Therefore, a precise LNP size control method is indispensable for understanding the effect of the LNP size on the DDS performance.

**Fig. 1:**
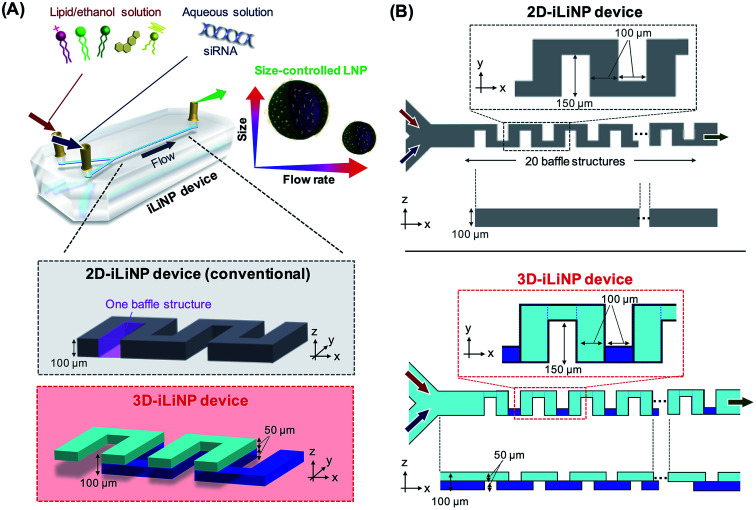
(A) Schematic and (B) top and cross-sectional views of the 2D- and 3D-iLiNP devices.

Solvent injection using microfluidic devices can control the LNP size more precisely compared to conventional batch scale production and size tuning methods.^10–13^ In the microfluidic-based LNP production method, the LNP size was controlled by the ethanol dilution rate. Therefore, the flow conditions, such as the total flow rate and flow rate ratio (FRR) of aqueous phase to lipid phase, and the microchannel geometry are key parameters for controlling the LNP size.^13–16^ To control the LNP size, the LNP formation behavior has been investigated using several types of microfluidic devices (Table S2†). The microfluidic hydrodynamic focusing device^17^ and the chaotic micromixer device^15^ are typical microfluidic devices for the production of LNPs with sizes of 50–60 and 30–60 nm, respectively. Although these LNPs are smaller than those produced with conventional methods, they do not cover the size range summarized in Table S1.† To address the LNP size controllability, we developed a microfluidic device named iLiNP™ (Fig. 1, 2D-iLiNP device).^14^ The iLiNP device was equipped with baffle structures and induced a generation of secondary flow at high total flow rate conditions. As a result, the iLiNP device achieved the production of LNPs in the average size range from 20 to 150 nm. However, the size distribution of large-sized LNPs needs to be improved to understand the influence of the LNP size on the DDS performance in detail.

Theoretically, the dilution rate and mixing homogeneity affect the LNP size and size distribution, respectively. Therefore, the chaotic micromixer device, which induces chaotic advection in the microchannel, allows the production of homogeneous-sized LNPs. In contrast, the 2D-iLiNP device allows the production of small- and large-size LNPs in the high and low flow rate conditions, respectively; the large-sized LNPs are formed through diffusion-based ethanol dilution. However, although fluid control is crucial for the improvement of the LNP size distribution, in particular for large-sized LNPs, the homogeneous and slow rate dilution is still challenging. From the viewpoint of fluid dynamics and mass transportation, the homogeneous and slow rate dilution is a competitive phenomenon to diffusion. To the best of our knowledge, the microfluidic device to achieve homogeneous and slow rate dilution for the improvement of the LNP size distribution has not been reported.

In this paper, we report a three-dimensional (3D), symmetrically assembled microchannel, named the 3D-iLiNP device, based on the 2D-iLiNP device. We designed the 3D baffle structures using computational fluid dynamics (CFD) simulations. We demonstrated the homogeneous and slow rate ethanol dilution by the generation of 3D secondary flows at the low flow rate condition. Finally, we investigated the effect of the LNP size distribution on the gene silencing activity *via in vitro* and *in vivo* experiments using siRNA-loaded LNPs.

## Results and discussion

### Design of the 3D-iLiNP device through computational fluid dynamics (CFD) simulations

**Fig. 2:**
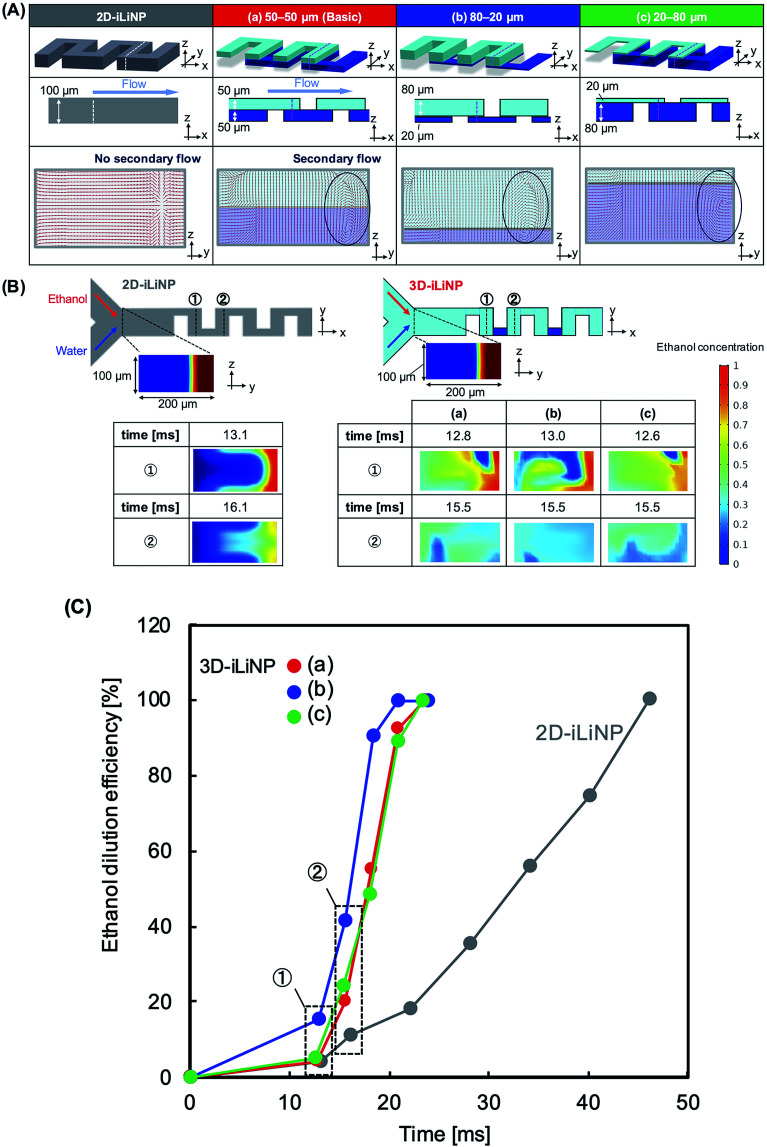
CFD simulation of ethanol dilution in the 2D- and 3D-iLiNP devices. The total flow rate was 50 μL min^−1^ and the FRR was 3. (A) Streamlines at the cross-section of the 2D- and 3D-iLiNP devices. The dashed line region in the 3D views represent the cross-section of the CFD results. The 3D-iLiNP devices consisted of top and bottom layers with different channel thickness; (a) 50–50 μm, (b) 80–20 μm, and (c) 20–80 μm. (B) Comparison of ethanol dilution process of the 2D- and 3D-iLiNP devices. (C) Ethanol dilution efficiency of the 2D-and 3D-iLiNP devices.

Fig. 1 shows a schematic of the 3D-iLiNP device equipped with 20 baffle structures based on the 2D-iLiNP device.^14^ With the 3D-iLiNP device, we predicted a secondary flow in the *x*–*z* direction in addition to that in the *y*–*z* direction at the 3D-baffle structures; this secondary flow would allow the acceleration of the ethanol dilution of the lipid solution. Fig. 2(A) shows the 3D views, cross-sectional views, and CFD results of the 2D- and 3D-iLiNP devices. As a proof of the concept experiment, we preliminarily designed the three-types of the 3D-iLiNP devices. The 3D-iLiNP devices consisted of top and bottom layers with different channel thickness; (a) 50–50 μm, (b) 80–20 μm, and (c) 20–80 μm. The total flow rate and the FRR (water to ethanol) was set to 50 μL min^−1^ and 3, respectively. We focused on the generation of the secondary flow in the 3D-baffle structures. As expected, the secondary circulation flow was generated at the baffle structures of the 3D-iLiNP devices, whereas no secondary flow was observed in the 2D-iLiNP device. Fig. 2(B) and (C) shows a comparison of the ethanol dilution process and ethanol dilution efficiency of the 2D- and 3D-iLiNP devices. The time differences to reach the solutions to cross-sections were shorter than 0.6 ms. In this FRR condition, the final ethanol concentration was 25%. The 2D-iLiNP device retained a high concentration of ethanol at the corner of the microchannel 16 ms after injection. The 3D-iLiNP devices diluted ethanol homogeneously compared with the 2D-iLiNP device. The 3D-iLiNP devices showed complete ethanol dilution within 20 ms, regardless of the device design (Fig. 2(C)). The design of the basic-type, 80–20 μm, and 20–80 μm 3D-iLiNP devices did not significantly affect the dilution performance of ethanol in the CFD simulation. The results of the CFD simulation confirm that the 3D-iLiNP device at a low total flow rate is a more suitable structure for homogeneous and slow rate ethanol dilution compared with the 2D-iLiNP device.^14,15^ This fluid dynamics is significant to control the LNP size at the low flow rate condition. Therefore, we carried out the evaluation of the LNP size controllability of the 3D-iLiNP devices.

### Evaluation of the LNP size controllability of the 3D-iLiNP devices

**Fig. 3:**
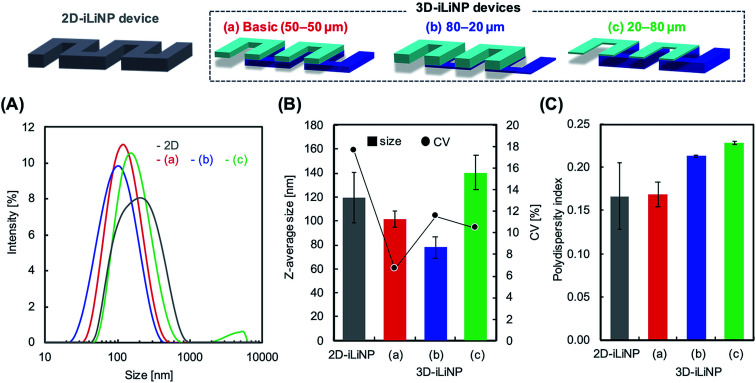
(A) Size distributions of the POPC LNPs prepared with the 2D- (gray) and 3D-iLiNP devices. The total flow rate was 50 μL min^−1^ and the FRR was 3. Red, blue, and green represent the (a) basic, (b) 80–20 μm, and (c) 20–80 μm devices, respectively. (B) Average sizes and coefficient variation (CV) values of the POPC LNPs. (C) Average values of the polydispersity index (PDI) of the POPC LNPs.

We determined the 3D-iLiNP device design with the highest LNP size controllability. Fig. 3(A) shows the size distribution of 1-palmitoyl-2-oleoyl-*sn*-glycero-3-phosphocholine (POPC) LNPs produced using the 2D and 3D-iLiNP devices, the basic-type, 80–20 μm, and 20–80 μm, at a total flow rate and FRR of 50 μL min^−1^ and 3, respectively. The basic-type 3D-iLiNP device showed the narrowest LNP size distribution among the four devices, the 2D and three types of 3D-iLiNP devices. Fig. 3(B) shows the average LNP size and coefficient variation (CV) value and Fig. 3(C) shows the polydispersity index (PDI) of the LNPs produced with the four devices. The average sizes of the POPC LNPs produced with the basic-type, 80–20 μm, and 20–80 μm 3D-iLiNP devices were 101, 78, and 141 nm, respectively. The CV values and PDI were approximately 7–12% and 0.17–0.23, respectively. In comparison with the 2D-iLiNP device, the basic-type 3D-iLiNP device showed small CV value and PDI even with a large particle size of approximately 100 nm. We assume the sharp change of the device geometry affects the fluid stability and LNP size controllability. From the viewpoint of microfluidics, two solutions introduce into a narrow microchannel is unstable compared with that of a wide microchannel due to the backpressure and the pressure balance of two solutions. As a result, the basic-type 3D-iLiNP device showed small CV value and PDI value. The relationship between the flow condition and the average LNP size is summarized in Fig. S2(A).† We also measured the concentrations of LNPs using a nanoparticle tracking analyzer (NS300, NanoSight, Malvern Instruments, Worcestershire, UK) (Fig. S2(B)†). The basic-type 3D-iLiNP device clearly improved the LNP size distribution. From these results, we decided to use the basic-type 3D-iLiNP device in this study.

**Fig. 4:**
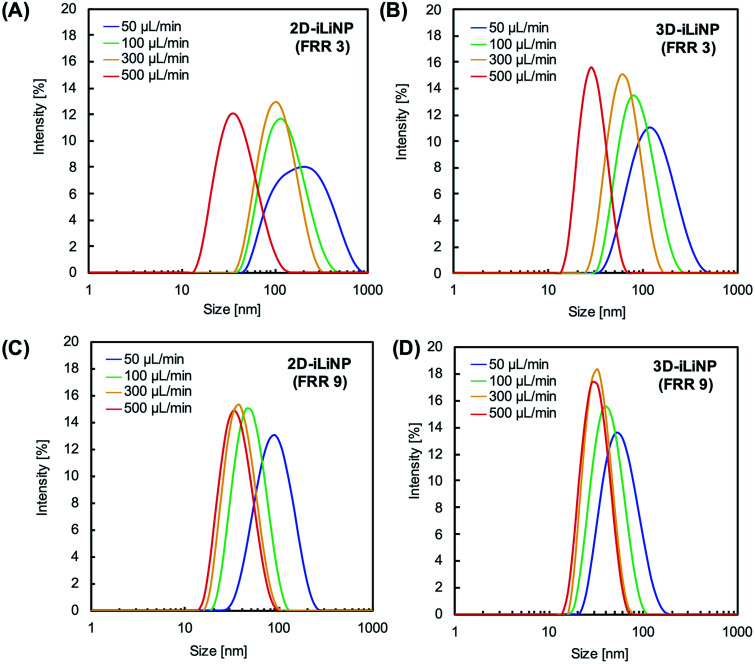
Size distributions of the POPC LNPs prepared at FRRs of (A and B) 3 and (C and D) 9. (A) and (C) 2D-iLiNP device; (B) and (D) 3D-iLiNP device.

**Fig. 5:**
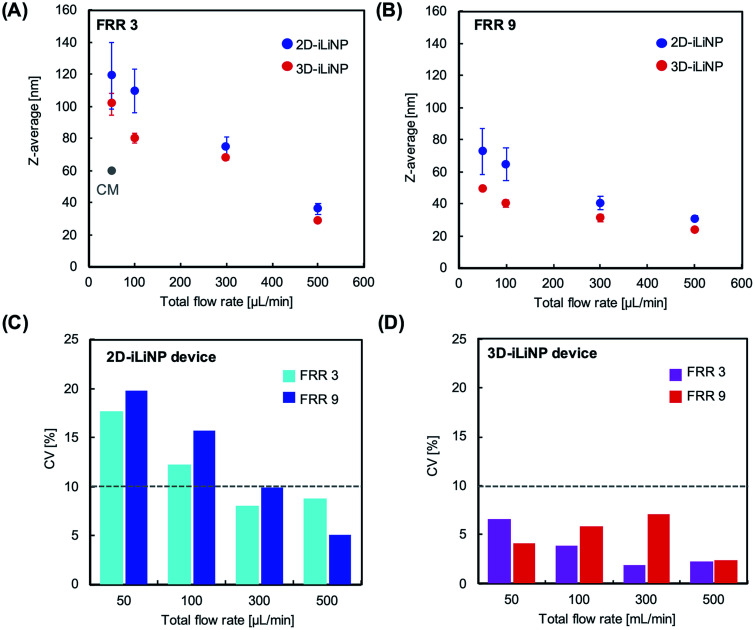
Average LNP size as a function of the total flow rate with an FRR of (A) 3 and (B) 9. (C, D) CV values of the LNPs prepared with the 2D- and 3D-iLiNP devices, respectively.

Fig. 4 shows the size distributions of the LNPs produced with the 2D- and 3D-iLiNP devices at total flow rates of 50 or 500 μL min^−1^ and FRRs of 3 and 9. In a previous study, it was reported that the increase in the total flow rate and FRR induces the production of small-sized LNPs.^14^ From Fig. 4, it can be seen that the size distribution of the LNPs produced using the 3D-iLiNP device in all flow conditions was significantly more homogeneous than that of the LNP produced with the 2D-iLiNP device. In particular, a single narrow peak can be observed in the size distribution of the LNPs produced with the 3D-iLiNP device at the flow rate of 50 μL min^−1^ and FRR of 3. Fig. 5(A) and (B) show a summary of the size of the LNPs produced with the 2D- and 3D-iLiNP devices with an FRR of 3 and 9, respectively. With an FRR of 3, the LNP size range of the iLiNP devices was approximately 30–100 nm, and the LNPs produced with the 3D-iLiNP device were slightly smaller than those produced with the 2D-iLiNP device. With an FRR of 9, the LNP size had a similar trend to that observed at an FRR of 3, and the size range of the LNPs produced with 3D-iLiNP device was 20–50 nm. By controlling the fluid flow, the size of the LNPs produced with the 3D-iLiNP device could be controlled with 10 nm precision (Fig. S3†), even when the device design was modified for mass production (Fig. S4†). Both the 2D- and 3D-iLiNP devices allowed the control of the LNP size through the control of the flow conditions; however, the 3D-iLiNP device led to LNPs with lower CV value in the low flow condition (Fig. 5(C) and (D)). In this study, we decide to 10% value of CV (for example, 20 nm ± 2 nm and 100 nm ± 10 nm) is a benchmark to evaluate the LNP size controllability.^16^ At all total flow values, the CV value of the LNPs produced with the 3D-iLiNP device was at least 7% smaller than that of the LNPs produced with the 2D-iLiNP device.

In the microfluidic-based LNP preparation method, the dilution rate affects the LNP size and the mixing homogeneity affects the LNP size distribution.^15^ From the viewpoint of fluid dynamics and mass transportation, it is difficult to achieve a homogeneous distribution and slow dilution rate because the homogeneous and slow rate dilution is a competitive phenomenon to diffusion. The concentration gradient induced by diffusion is the major reason for the formation of heterogeneous-sized LNPs because ethanol dilution is a trigger for LNP formation. Therefore, a microfluidic device suitable for low flow rate conditions is indispensable for producing homogeneous- and large-sized LNPs. On the other hand, a homogeneous size distribution and rapid dilution can be achieved with several types of microfluidic devices, such as the chaotic micromixer and 2D-iLiNP devices. The chaotic micromixer device allows homogeneous mixing under low flow rates;^18^ however, it could not produce the large-sized LNPs (Table S2†).^15^ In the case of the 2D-iLiNP device, the formation of the secondary flow plays an important role in the LNP size controllability and depends on the total flow rate.^14^ The 2D-iLiNP device allows the control of the LNP Z-average size in the wide range of 20–120 nm through the flow condition. However, the formation of the secondary flow at the low flow rate condition in the 2D-iLiNP was not sufficient to produce homogeneous- and large-sized LNPs. In this study, we revealed that the homogeneous and slow rate dilution was compatible with the rapid dilution by the 3D-iLiNP device, which allowed the production of LNPs in the size range of 20–100 nm with low CV values.

### Application for siRNA-LNP systems

**Fig. 6:**
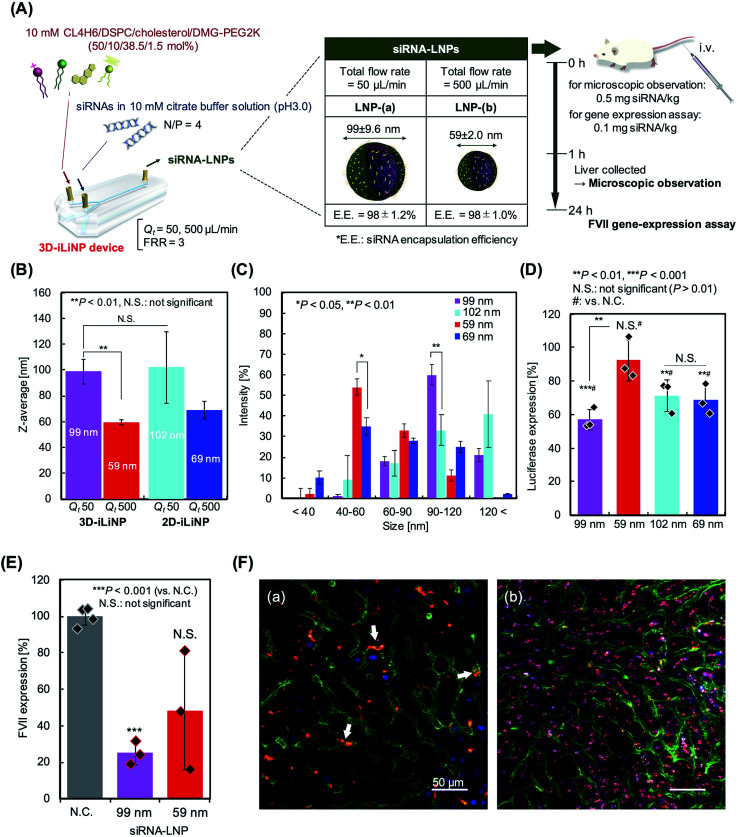
(A) Schematic of the CL4H6-based LNP preparation and *in vivo* experimental design. (B) Average size of the CL4H6 LNPs prepared with the 3D and 2D-iLiNP devices. **P < 0.01, N.S.: not significant (*P* > 0.05), *n* = 3, Student's *t*-test. (C) Size distributions of the CL4H6 LNPs. *P < 0.05, **P < 0.01, *n* = 3, Student's *t*-test. (D) Gene-silencing activity of the 99 and 59 nm LNPs in HeLa-dluc cells at a dose of 0.1 nM siGL4. **P < 0.01, ***P < 0.001 (#: *vs.* N.C.), N.S.: not significant (#: *vs.* N.C., *P* > 0.01), *n* = 3, Student's *t*-test. (E) FVII expression of the ICR mice treated with CL4H6 LNPs at a dose of 0.1 mg siRNA/kg. ***P < 0.001 (*vs.* N.C.), N.S. not significant (*P* > 0.01), *n* = 3–4, Student's *t*-test. (F) Confocal microscope images of the mice liver tissues treated with (a) 99 and (b) 59 nm LNPs at a dose of 0.5 mg siRNA per kg. Blue, green, red, and cyan represent nuclei, blood vessel, LNP, and siRNA, respectively. The arrows indicate the accumulation of LNPs in the blood vessels.

We applied the 3D-iLiNP device for siRNA-encapsulated LNP production to investigate the effect of the LNP size distribution on the gene silencing activity in *in vitro* and *in vivo* experiments. Our original synthesized pH-sensitive cationic lipid, named CL4H6,^19^ and 1,2-dioleoyl-3-dimethylammonium-propane (DODAP), a commercially available lipid, were used as the main components of the lipid systems. Fig. 6(A) shows a schematic of the CL4H6-based LNP preparation process and *in vivo* experimental design. At total flow rates of 50 or 500 μL min^−1^, the 3D-iLiNP device produced 99 and 59 nm LNPs (Fig. 6(B)). At the same total flow rates, the average size of the CL4H6 LNPs produced with the 2D-iLiNP device were 102 and 69 nm, respectively. The siRNA encapsulation efficiency of the LNPs was higher than 97% (Table S3†). The CV values of the LNPs produced with the 3D-iLiNP device were lower than 10%, while the PDI was almost the same among the four types of LNPs. We did not observe a statistically significant difference on the average size of the 99 and 102 nm-sized LNPs. To investigate the effect of the LNP size distribution on the gene silencing activity, we classified the LNP size into five size ranges based on the LNP size distribution (Fig. 6(C) and S5†): <40, 40–60, 60–90, 90–120, and >120 nm. From the LNP size distribution, we confirmed that the 40–60 and 90–120 nm LNPs were the main size ranges of the 59 and 99 nm sized LNPs prepared with the 3D-iLiNP device, respectively. The intensity of the main peaks of both LNPs at the two size ranges were higher than 50%. In contrast, 40–60 and >120 nm were the main size ranges of the 69 and 102 nm LNPs, respectively; however, the intensity of the main peaks of both particles at the two size ranges were lower than 50%. In particular, the intensity of the 99 nm LNPs in the 90–120 nm size range was twice as high as that of the 102 nm LNPs.

Next, we carried out an *in vitro* experiment to evaluate the gene silencing activity of the LNPs with four sizes (Fig. 6(D)). The 99 nm LNPs showed the best gene silencing activity among the four LNP sizes and suppressed almost 40% of the luciferase expression at a dose of 0.1 nM siRNA. Interestingly, the 102 and 69 nm LNPs prepared with the 2D-iLiNP device suppressed luciferase expression by 30%, whereas the 59 nm LNPs prepared with the 3D-iLiNP device did not induce gene silencing. It was found that gene silencing activity depends on the amount of LNPs with size ranging from 90 to 120 nm. The DODAP LNPs showed a similar trend to that of the CL4H6 LNPs (Fig. S6†). This result indicates that the LNP size distribution plays a crucial role on the gene silencing activity, and 3D-iLiNP allows the production of LNPs highly efficient for siRNA delivery. Fig. 6(E) shows the gene silencing activity observed in the *in vivo* experiment. The lipid system was optimized for the *in vivo* experiment;^19^ therefore, both the 99 and 59 nm LNPs suppressed plasma coagulation factor VII (FVII) expression in mice at a dose of 0.1 mg siRNA per kg. The 99 nm LNPs showed 75% gene silencing activity, while the 59 nm LNPs suppressed 50% of the FVII expression. Fig. 6 (F) shows the intrahepatic LNP distributions of the 99 and 59 nm LNPs. We observed a size-specific intrahepatic LNP distribution consistent with previous reports.^3^ From the results of the gene silencing activity experiments, intrahepatic distribution, and size of fenestrae (approximately 100–150 nm), the LNPs with a size range of 90–120 nm exhibited the highest RNA delivery performance *in vivo* using this lipid system. Therefore, the 3D-iLiNP device will be a key technology for the production of size-controlled LNPs for RNA delivery applications.

## Conclusion

We developed a 3D, symmetrically assembled microfluidic device named 3D-iLiNP with the aim of improving the size distribution of large-sized LNPs produced at low flow rate conditions. The 3D-iLiNP device allowed the production of LNPs with a narrow size distribution owing to the 3D secondary flow. We demonstrated that the 3D-iLiNP device could produce POPC LNPs in the size range of 20 to 100 nm depending on the flow conditions with CV values smaller than 10%. We employed the 3D-iLiNP device for the production of siRNA-loaded LNPs to investigate the effect of the LNP size distribution on the gene silencing activity. The 3D-iLiNP device allowed the production of siRNA-loaded LNPs with a size distribution more homogenous than that of the LNPs produced with the 2D-iLiNP device. We clearly distinguished the main LNP size ranges produced by the 3D-iLiNP device and found that the 90–120 nm LNPs suppressed the luciferase expression of HeLa cells. For the *in vivo* experiment, 100 nm LNPs showed a higher gene silencing activity than the small-sized siRNA LNPs. We believe that the 3D-iLiNP device and size-controlled LNPs could improve not only the RNA delivery and gene silencing activity, but also the transfection or genome-editing performance of LNP-DDSs.

## Experimental procedures

### Materials

1-Palmitoyl-2-oleoyl-*sn*-glycero-3-phosphocholine (POPC), 1,2-distearoyl-*sn*-glycero-3-phosphocholine (DSPC), 1,2-dioleoyl-*sn*-glycero-3-phosphoethanolamine (DOPE), 1,2-dioleoyl-3-dimethylammonium-propane (DODAP), and 1,2-dimyristoyl-rac-*glycero*-3-methoxypolyethylene glycol-2000 (DMG-PEG2K) were purchased from the NOF Corporation (Tokyo, Japan). Cholesterol was purchased from Sigma-Aldrich (St. Louis, MO, USA). A pH-sensitive cationic lipid, CL4H6, was synthesized in the laboratory as previously described.^19^ Ethanol, sodium chloride, citric acid, 2-morpholinoethanesulfonic acid (MES), monohydrate, and phosphate-buffered saline (PBS) were purchased from FUJIFILM Wako Pure Chemical Corporation (Osaka, Japan). The siFVII and Cy-5-labeled siGL4 were purchased from Hokkaido System Science Co. Ltd. (Sapporo, Japan). Table S4† shows the sense and antisense strand sequences of siFVII and Cy5-siGL4. Quant-iT™ Ribogreen™ RNA reagent and 1,1′-dioctadecyl-3,3,3′,3′-tetramethylindocarbocyanine perchlorate (DiI) were obtained from Thermo Fisher Scientific (Waltman, MA, USA). SU-8 3050 was obtained from Nippon Kayaku Co., Ltd. (Tokyo, Japan).

### Computational fluid dynamics study

COMSOL Multiphysics 5.2 (COMSOL, Inc., Burlington, MA) was used for a CFD study. The experimental conditions were the same as those reported in a previous study.^14^ The profiles of the ethanol concentration were numerically simulated using 3D models under both the laminar-flow model (no slip) and transport of diluted species (ethanol) condition. The flow was modeled as an incompressible flow using the Navier–Stokes equation. The total flow rate and FRR (water/ethanol) were set to 50 μL min^−1^ and 3, respectively. For the calculation of the ethanol dilution efficiency, we counted a number of mesh with 20% ethanol concentration at each cross-section and each residence time.^20^ Then, the ethanol dilution efficiency was calculated from the following equation.Ethanol dilution efficiency [%] = number of meshes with 20% ethanol concentration/total number of meshes × 100

### Fabrication of iLiNP devices

We made poly(dimethylsiloxane) (PDMS) replicas for the 2D- and 3D-iLiNP devices using the standard photolithography method.^21^ For the alignment of the 3D-iLiNP device, we employed amino silane coupling with minor modifications (Fig. S1†).^22^

### Preparation of LNPs using the 3D-iLiNP device

POPC was used as a model LNP to investigate the size controllability of the 3D-iLiNP device. POPC LNPs were prepared using a 13.4 mM POPC/ethanol solution and saline. The collected LNP suspensions were dialyzed against saline overnight.

To validate the applicability of the 3D-iLiNP device for the production of siRNA-loaded LNPs, we investigated DODAP- and CL4H6-based LNP systems. The lipid amine-to-oligonucleotide phosphate (N/P) ratio was fixed to 4. The siRNA-loaded DODAP LNPs were prepared by mixing an 8 mM lipid/ethanol solution composed of DODAP/DOPE/cholesterol/DMG-PEG 2K (30/25/40/5 mol%) with a 10 mM citrate buffered solution (pH3.0) containing siRNAs at a concentration of 47.5 μg mL^−1^. The siRNA-loaded CL4H6 LNPs were prepared by mixing a 10 mM lipid/ethanol solution composed of CL4H6/DSPC/cholesterol/DMG-PEG 2K (50/10/38.5/1.5 mol%)^23^ with a 10 mM citrate buffered solution containing siRNAs at a concentration of 132 μg mL^−1^. The lipid solution and siRNA in the buffer solution were separately introduced into the iLiNP devices at a total flow rates of 50 or 500 μL min^−1^ and FRR of 3. The collected LNP suspensions were dialyzed against 20 mM MES buffer solution (pH 6.0) for 2 h followed by an overnight dialysis against PBS. The siRNA encapsulation efficiency was measured using a Ribogreen assay.^3^ For *in vivo* experiments, the CL4H6-LNP suspensions were concentrated using Amicon Ultra filters (100K, Merck, Darmstadt, Germany).

The size and polydispersity index (PDI) of the LNPs were measured by dynamic light scattering (DLS) measurements obtained with a Zetasizer Nano ZEN3600 (Malvern Instruments, Worcestershire, U.K.).

### *In vitro* assay

HeLa cells stably expressing Firefly and Renilla luciferase (HeLa-dluc) were cultured in the same conditions as described previously.^19^ Cells were seeded at a concentration of 3 × 10^3^ and 6 × 10^3^ cells per well in a 96 well-microplate for 24 h prior to the LNP treatment for the cell viability assay and gene expression assay. Then, the cells were treated with LNPs and incubated for 24 h. After the incubation, the cell viability was measured using a Cell Counting Kit-8 (Dojindo, Kumamoto, Japan) according to the manufacture's protocol. For gene expression assay, firefly and renilla luciferase activities were measured using a Dual-Glo assay and a GloMax®Explorer System (Promega), according to the manufacture's protocol.

### *In vivo* experiment

The gene knockdown activity of coagulation factor VII (FVII) and the confocal microscope images of the mice's hepatocytes were analyzed following previously described protocols^14^ with minor modifications. For the observation of the intrahepatic siRNA distributions, we prepared DiI- and (Cy5)-labeled siGL4 encapsulated CL4H6 LNPs by mixing a 10 mM lipid/ethanol solution containing 0.5 mol% DiI with a 10 mM citrate buffered solution containing Cy5-labeled siGL4. All animal procedures were performed in accordance with the Guidelines for Care and Use of Laboratory Animals of Hokkaido University and approved by the Hokkaido University Animal Ethics Committee.

### Evaluation of scale-up performance of the 3D-iLiNP device

The 3D-iLiNP device used for the investigation of the mass-produced LNPs was made from cycloolefin polymer plates fabricated by a micromachining process (Zeon Corporation, Tokyo, Japan).

### Statistical analysis

The results were analyzed with Microsoft® Excel and an unpaired Student's *t*-test was used to compare the average values of two groups.

## Author contributions

Conceptualization: M. M. and M. T.; data curation: N. K. and M. M.; formal analysis: N. K., M. M., Y. S, A. I., H. T., H. H., and M. T.; methodology: M. M. and M. T.; investigation: N. K., M. M., K. S., and Y. S.; writing-original draft: N. K. and M. M.; writing-review and editing: M. M. and M. T.; funding acquisition: N. K., M. M., H. H., and M. T.; resources: N. K., M. M., and Y. S.; supervision: M. M. and M. T.

## Conflicts of interest

The authors declare no competing financial interest.

## Supplementary Material

RA-011-D0RA08826A-s001
