# Supplementary material for: Three-dimensional, symmetrically assembled microfluidic device for lipid nanoparticle production
Source: RSC Adv. 2021 Jan 5;11(3):1430–9. doi: 10.1039/d0ra08826a (PMC8693528; doi:10.1039/d0ra08826a)
Supplement: RA-011-D0RA08826A-s001 [file RA-011-D0RA08826A-s001.pdf]

Electronic Supplementary Material (ESI) for RSC Advances.
This journal is © The Royal Society of Chemistry 2020

**Supporting Information**

# Three-Dimensional, Symmetrically Assembled Microfluidic Device for Lipid Nanoparticle Production

*Niko Kimura,[a#] Masatoshi Maeki,[b,c#*] Yusuke Sato,[d] Kosuke Sasaki,[d] Akihiko Ishida,[b] Hirofumi Tani,[b] Hideyoshi Harashima,[d] and Manabu Tokeshi[b,e,f*]*

*[a]Graduate School of Chemical Sciences and Engineering, Hokkaido University, Kita 13 Nishi 8, Kita-ku, Sapporo, 060-8628, Japan*

*[b]Division of Applied Chemistry, Faculty of Engineering, Hokkaido University, Kita 13 Nishi 8, Kita-ku, Sapporo 060-8628, Japan*

*[c]JST PRESTO, 4-1-8 Honcho, Kawaguchi, Saitama, 332-0012, Japan*

*[d]Faculty of Pharmaceutical Sciences, Hokkaido University, Kita 12 Nishi 8, Kita-ku, Sapporo 060-0812, Japan*

*[d]Innovative Research Center for Preventive Medical Engineering, Nagoya University, Furo-cho, Chikusa-ku, Nagoya 464-8601, Japan*

*[e]Institute of Nano-Life Systems, Institutes of Innovation for Future Society, Nagoya University, Furo-cho, Chikusa-ku, Nagoya 464-8601, Japan*

## 1. Fabrication of the 3D-iLiNP device

We employed amino silane coupling with minor modification.[1] We modified a concentration of 3-aminopropyltriethoxysilane (APTES, Tokyo Chemical Industry, Japan) solution form 1% v/v to 3% v/v. Replicated PDMS pieces were treated with oxygen plasma (CUTE-1MP/R, Femto Science, Gwangju, Korea) and soaked in 3%v/v APTES aqueous solution for 20 min at room temperature. Then, the silanized replicas were washed with DI water and aligned with each replica using an optical microscope. After bonding the replicas, the amino groups remaining on the surface of the microchannel were acetylated by introducing 20%v/v acetic anhydride (Tokyo Chemical Industry, Japan) containing dimethylformamide solution.

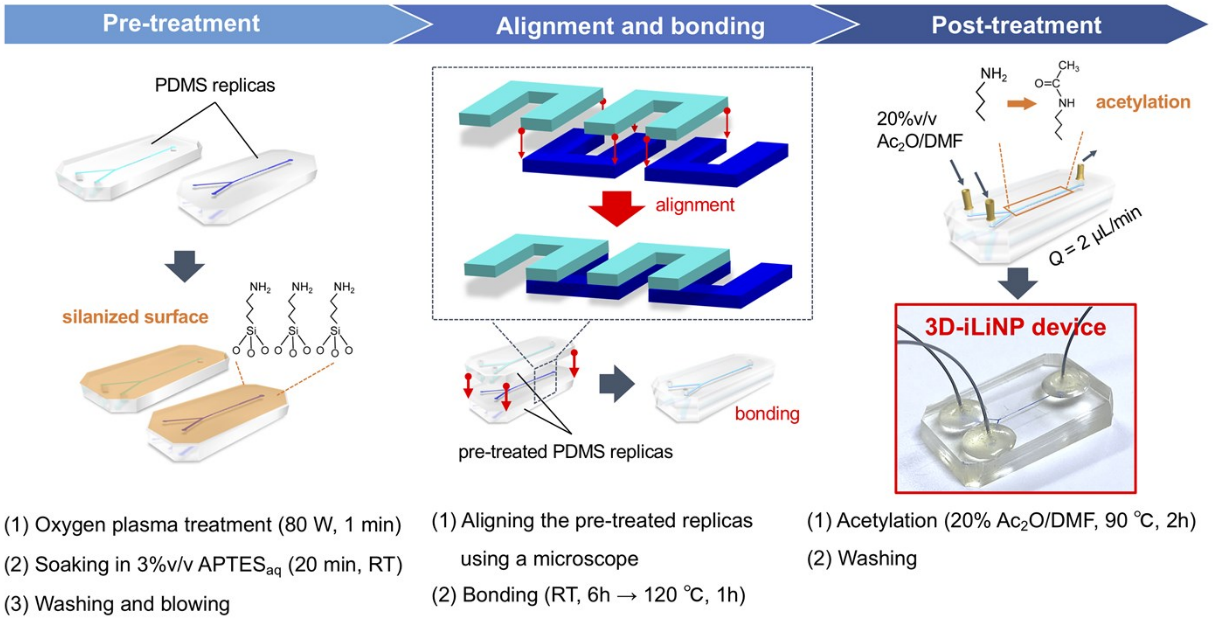


Figure S1. Schematic of the 3D-iLiNP device fabrication process

## 2. Evaluation of the LNP size controllability of the 3D-iLiNP devices

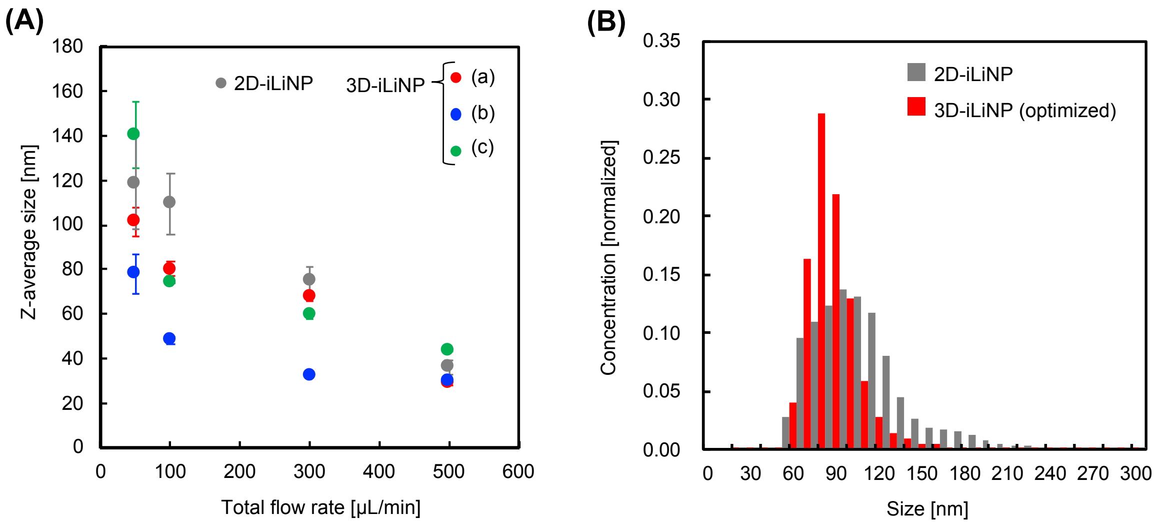


Figure S2. Relationship between the flow condition and the average LNP size. The LNP size was measured by (A) a Zetasizer Nano ZEN3600 and (B) a nanoparticle tracking analyzer. The LNPs measured by the nanoparticle tracking analyzer were prepared using the 2D-iLiNP (gray) and the 3D-iLiNP devices (red) at total flow rate of 50 µL/min and an FRR of 3.

### 3. Control of LNP size with 10 nm intervals through the flow conditions

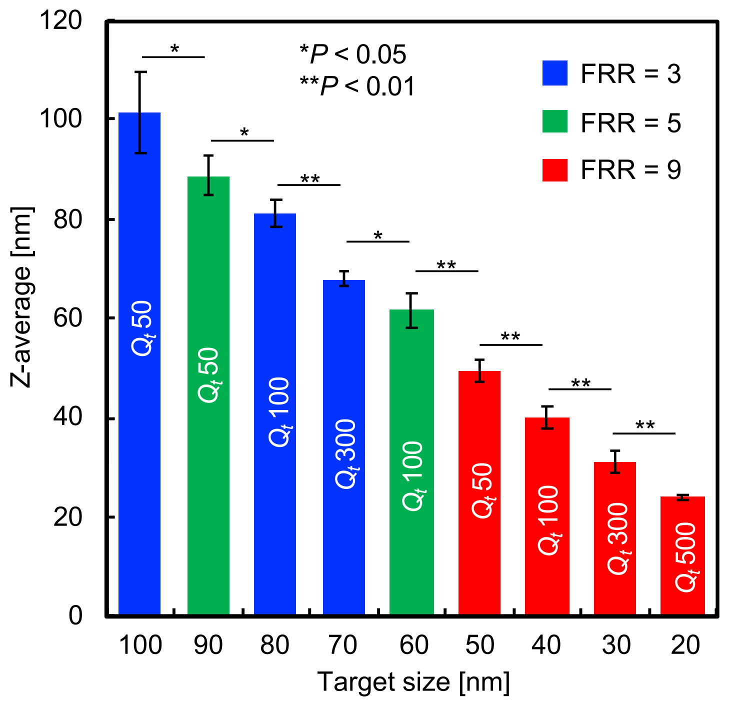


Figure S3. Control of the POPC LNP size with 10 nm intervals using the 3D-iLiNP device. The total flow rate was 50–500 µL/min and the FRR was 3–9. *$P < 0.05$, **$P < 0.01$, Student's t-test, $n = 3–4$.

## 4. Scale-up performance of the 3D-iLiNP device

The 3D-iLiNP device for the large-scale LNP production was made from cycloolefin polymer plates fabricated by a micromachining process (Zeon Corporation, Tokyo, Japan). To prepare the LNPs, 13 mM POPC/ethanol solution and saline were introduced into the device. The total flow rate was 500–5000 µL/min and the FRR was 3 or 9.

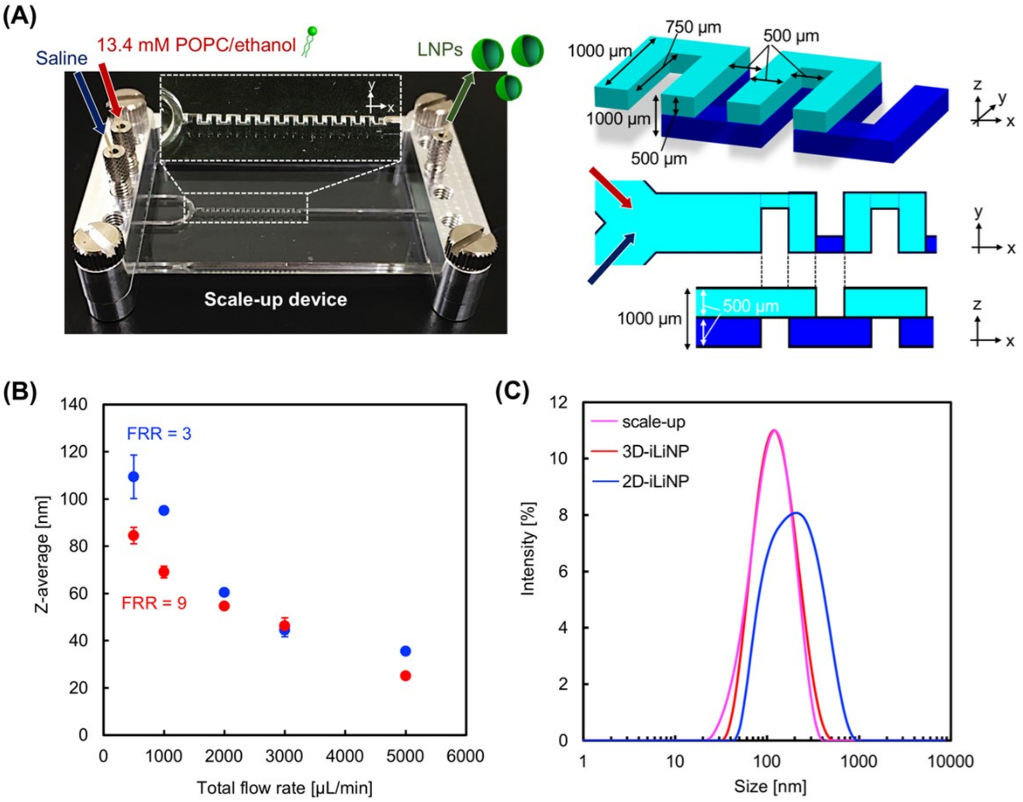


Figure S4. (A) Photograph of the 3D-iLiNP device for the scale-up of the LNP production. (B) Relationship between the POPC LNP sizes and total flow rate. (C) Size distributions of 100 nm-sized POPC LNPs prepared using the 3D- (scale-up: magenta; basic: red) and 2D-iLiNP devices (blue). The total flow rate was 50 µL/min and the FRR was 3.

## 5. Application of 3D-iLiNP for siRNA-LNP systems

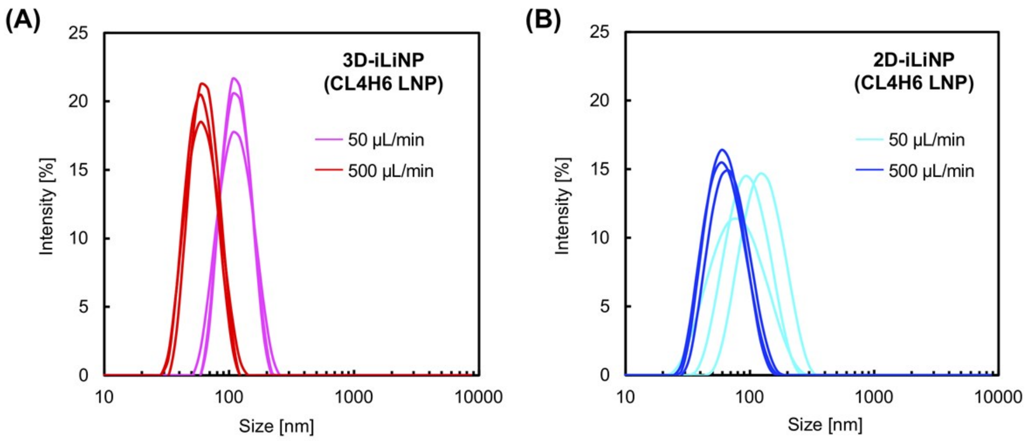


Figure S5. Size distributions of siRNA-encapsulated CL4H6 LNPs prepared with the (A) 3D- and (B) 2D-iLiNP devices. The total flow rate was 50 or 500 μL/min and the FRR was 3.

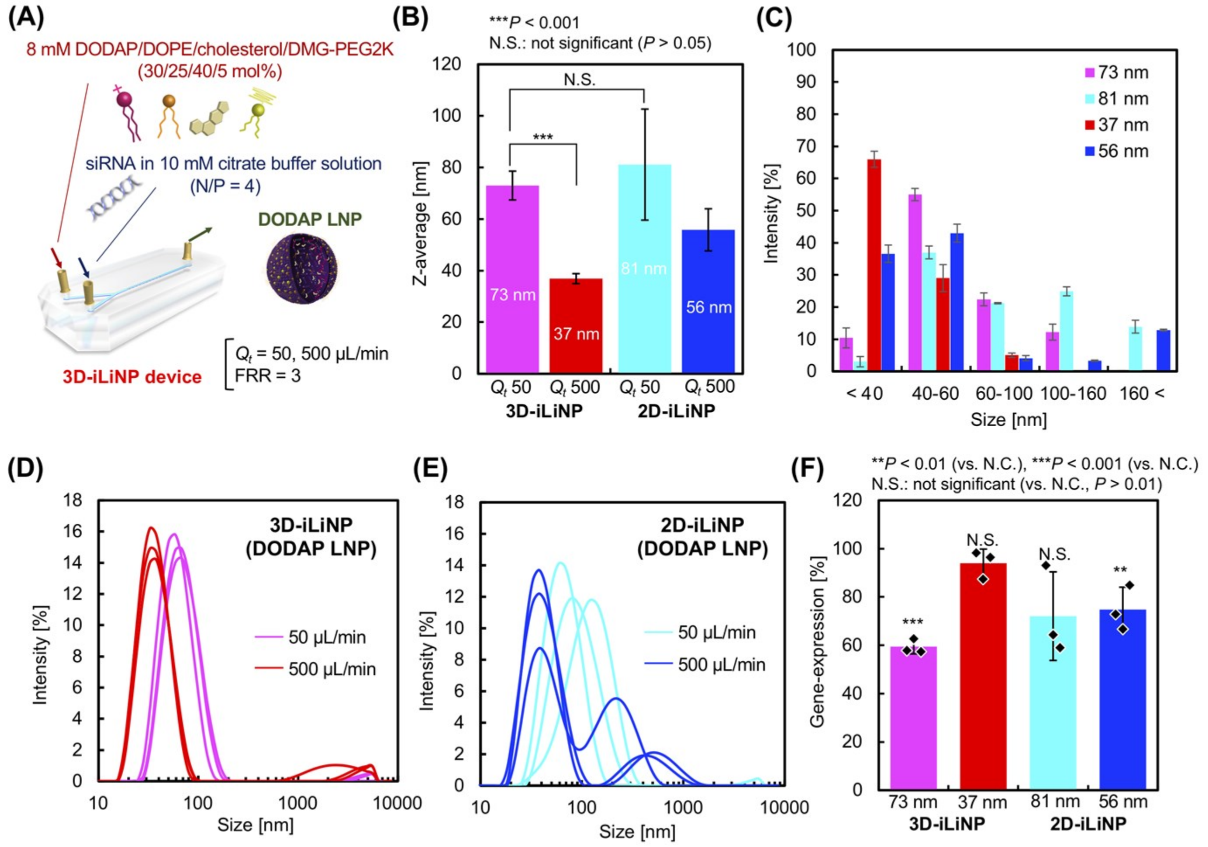


Figure S6. (A) Schematic of the DODAP-based LNP preparation. (B) Z-average sizes and (C) size distributions of the DODAP LNPs. The LNP sizes were classified into five size ranges (< 40, 40–60, 60–100, 100–160, and >160) based on the size distributions of the LNPs produced with the (D) 3D- and (E) 2D-iLiNP devices. The total flow rate was 50 or 500 µL/min and the FRR was 3. ***$P < 0.001$, N.S.: not significant ($P > 0.05$), Student's t-test, $n = 3$. (F) Gene-silencing activity of the LNPs at a dose of 50 nM siGL4. **$P < 0.01$, ***$P < 0.001$, N.S.: not significant ($P > 0.01$), Student's t-test, $n = 3$.

## Supplemental Tables

Table S1. Relationship between the LNP size and DDS in vivo according to different studies

| Reference | | Year | LNP system | Size and DDS in vivo |
|---|---|---|---|---|
| [1] | Mukhamadiyarov, R. A. *et al.* | 2018 | EPC/cholesterol | 70 nm > 100 nm accumulation at rats' ischemic myocardium |
| [2] | Cabral, H. *et al.* | 2011 | (PEG-*b*-P(Glu)) copolymer | 30 nm > 50 nm antitumor activity in pancreatic tumors |
| [3] | Sato, Y. *et al.* | 2016 | YSK05/cholesterol/DMG-PEG 2K | 30 nm > 50 nm siRNA delivery to mice's liver tissues |
| [4] | Hirsjarvi, S. *et al.* | 2013 | Solutol®/Lipoid®/Labrafac® | 25 nm > 50 nm rapid distribution to peripherical capillaries in mice's tissues |
| [5] | Ren, H. *et al.* | 2019 | EPC/cholesterol/DSPE-PEG 2K | 100 nm > 70 nm rheumatoid arthritis targeting in mice |
| [6] | Chen, S. *et al.* | 2016 | DMAP-BLP/DSPC/cholesterol/DMG-PEG 2K | 80 nm > 30 nm FVII gene-silencing in mice |
| [7] | Yanez Arteta, M. *et al.* | 2018 | DLin-MC3-DMA/DSPC/cholesterol/DMPE-PEG 2K | 65 nm > 50 nm mRNA delivery to mice's hepatocytes and protein expression |

PEG-*b*-P(Glu): poly(ethylene glycol)-*b*-poly(glutamic acid), EPC: ethyl phosphatidylcholine, DMG-PEG 2K: 1,2-dimyristoyl-rac-glycero-3-[methoxy(polyethyleneglycol)-2000], DSPE-PEG 2K: distearoyl phosphatidylethanolamine-[methoxy(polyethyleneglycol)-2000], DSPC: 1,2-distearoyl-sn-glycero-3-phosphocholine, DMAP-BLP: 3- (dimethylamino)propyl(12Z,15Z)-3-[(9Z,12Z)-octadeca-9,12-dien-1-yl]henicosa-12,15-dienoate, DMPE-PEG 2K: 1,2-dimyristoyl-sn-glycero-3- phosphoethanolamine-N-[methoxy(polyethylene glycol)-2000]

Table S2. Sizes of POPC LNPs prepared by using the several microfluidic devices

| Reference | | Year | Microfluidic device | Average size | FRR | Total flow rate |
|---|---|---|---|---|---|---|
| [12] | Mijajlovic, M. *et al.* | 2013 | MHF-device | 50-60 nm | 20-40 | 126-246 μL/min |
| [13] | Maeki, M. *et al.* | 2017 | CM device | 30-60 nm | 3-9 | 50-500 μL/min |
| [14] | Kimura, N. *et al.* | 2018 | iLiNP device | 20-150 nm | 3-9 | 50-500 μL/min |

MHF: microfluidic hydrodynamic focusing, CM: chaotic micromixer, iLiNP: invasive lipid nanoparticle production

Table S3. Summary of siRNA LNPs characteristics.

| pH-sensitive cationic lipid | Device | Total flow rate [μL/min] | Z-average [nm] | CV [%] | PDI | E.E.[a] [%] |
|---|---|---|---|---|---|---|
| DODAP | 3D | 50 | 73±5.6 | 8 | 0.15±0.012 | 98±1.0 |
| | | 500 | 37±1.9 | 5 | 0.22±0.040 | 97±1.5 |
| | 2D | 50 | 81±21.5 | 27 | 0.16±0.015 | 98±1.0 |
| | | 500 | 56±8.2 | 15 | 0.30±0.093 | 98±1.0 |
| CL4H6 | 3D | 50 (LNP-(a)) | 99±9.6 | 10 | 0.09±0.010 | 98±1.2 |
| | | 500 (LNP-(b)) | 59±2.0 | 3 | 0.07±0.037 | 98±1.0 |
| | 2D | 50 | 102±27.7 | 27 | 0.13±0.039 | 97±2.5 |
| | | 500 | 69±6.8 | 10 | 0.16±0.086 | 98±1.8 |

[a]E.E.: Encapsulation efficiency

Table S4. Sequences of siRNAs

| siRNA | | Sequence |
|---|---|---|
| siFVII | sense | 5′-GGAucAucucAAGucuuAcTsT-3′ |
| | antisense | 5′-GuAAGAcuuGAGAuGAuccTsT-3′ |
| Cy5-siGL4 | sense | Cy5-CCGUCGUAUUCGUGAGCAATsT-3′ |
| | antisense | 5′-UUGCUCACGAAUACGACGGTsT-3′ |

**References**

References

1. L. Tang and N. Y. Lee, *Lab Chip*, 2010, **10**, 1274-1280.
